# Aggressive treatment with noninvasive ventilation for mild acute hypoxemic respiratory failure after cardiovascular surgery: Retrospective observational study

**DOI:** 10.1186/1749-8090-7-41

**Published:** 2012-05-03

**Authors:** Keiko Nakazato, Shinhiro Takeda, Keiji Tanaka, Atsuhiro Sakamoto

**Affiliations:** 1 Department of Anesthesiology, Nippon Medical School, 1-1-5 Sendagi, Bunkyo-ku, Tokyo, 113-8603, Japan; 2 Division of Intensive Care Unit and Coronary Care Unit, Nippon Medical School Hospital, 1-1-5 Sendagi, Bunkyo-ku, Tokyo, 113-8603, Japan

**Keywords:** Acute hypoxemic respiratory failure, Cardiovascular surgery, Post-extubation, Noninvasive ventilation, PaO_2_/FIO_2_

## Abstract

**Background:**

Acute hypoxemic respiratory failure (AHRF) is one of the most serious complications after cardiovascular surgery. It remains unclear whether noninvasive ventilation (NIV) has potential as an effective therapy for AHRF after cardiovascular surgery, although many reports have described the use of NIV for AHRF after extubation. The aim of this study was to investigate the effectiveness of NIV in the early stage of mild AHRF after cardiovascular surgery.

**Methods:**

We retrospectively analyzed all patients admitted to the intensive care unit after cardiovascular surgery, whose oxygenation transfer (PaO_2_/FIO_2_) deteriorated mildly after extubation, and in whom NIV was initiated. A two-way analysis of variance and the Bonferroni multiple comparisons procedure, the Mann–Whitney test, Fisher’s exact test or the *χ*^*2*^test was performed.

**Results:**

A total of 94 patients with AHRF received NIV, of whom 89 patients (94%) successfully avoided endotracheal intubation (successful group) and five patients required reintubation (reintubation group). All patients, including the reintubated patients, were successfully weaned from mechanical ventilation and discharged from the intensive care unit. In the successful group, PaO_2_/FIO_2_ improved and the respiratory rate decreased significantly within 1 h after the start of NIV, and the improvement in PaO_2_/FIO_2_ remained during the whole NIV period.

**Conclusion:**

We conclude that NIV is beneficial for mild AHRF after cardiovascular surgery when it is started within 3 h after mild deterioration of PaO_2_/FIO_2_. We also think that it is important not to hesitate before performing reintubation when NIV is judged to be ineffective.

## Background

Acute hypoxemic respiratory failure (AHRF) is one of the most serious complications after cardiovascular surgery, and postoperative pulmonary complications, which are as common as cardiac complications, prolong the hospital stay and increase the mortality rate [1]. In a review, the average postoperative values were reported to be very high for the incidences of atelectasis (15–98%) and pneumonia (0–20%) [2].

Noninvasive ventilation (NIV) can be used in postoperative patients. In a randomized study after elective major abdominal surgery, AHRF patients who received continuous positive airway pressure (CPAP) had significantly lower rates of reintubation(1% vs 10%) and occurrences of pneumonia, infection, and sepsis than patients treated with oxygen alone, and spent significantly fewer mean days in the intensive care unit (ICU) than patients treated with oxygen alone [3]. Furthermore, NIV is effective for AHRF following extubation after cardiac surgery [4]. The application of CPAP was reported to be associated with fewer pulmonary complications (PaO_2_/FIO_2_ < 100 mmHg, atelectasis, pneumonia, reintubation rate (4% vs 16%)) compared with the control group, and the mean length of hospital stay was significantly shorter in the CPAP group among patients who underwent elective prosthetic replacement of the thoracoabdominal aorta [4]. On the other hand, NIV did not prevent the need for reintubation or reduce mortality for unselected AHRF patients after extubation in some reports. In a multicenter randomized trial for AHRF patients after extubation, there was no difference in the need for reintubation (48% vs 48%) between the NIV group and the standard therapy group, and the mortality in the ICU was higher in the NIV group than in the standard therapy group [5]. In another randomized study for AHRF patients after extubation, there was no difference in the rates of reintubation (72% vs 69%) or hospital mortality between the NIV group and the standard medical therapy group, and similarly no difference was found in the duration of mechanical ventilation or length of ICU or hospital stay [6].

New potential indications of NIV will also be discussed, such as prophylactic NIV, especially in the postoperative or post-extubation period. Although the treatment procedure of AHRF after cardiac surgery remains unclear, we think that aggressive use of NIV for the early stage of mild AHRF is more effective at reducing reintubation rates and mortality. Further studies are needed for this situation before NIV can be regarded as a first-line treatment [7].

The aim of this study was to investigate the effectiveness of NIV in the early stage of mild AHRF after cardiovascular surgery.

## Methods

We retrospectively analyzed all patients who were admitted to the ICU of Nippon Medical School Hospital after cardiovascular surgery from April 2007 to March 2011. We conducted a retrospective and observational study of all AHRF patients after cardiovascular surgery in whom NIV was used in the ICU as initial ventilatory support. The ethical committee in our hospital has established that a retrospective study involving research of medical records can be conducted without authorization from the committee.

AHRF was defined according to a randomized study [3]. The indications for NIV in AHRF patients were determined by the physicians, and required the following clinical or physiologic criteria: oxygenation transfer deterioration (PaO_2_/FIO_2_ ≤ 300 mmHg); severe respiratory distress with dyspnea; respiratory rate > 20 breaths/min; typical findings on chest radiograph; use of accessory muscles; or extensive diaphoresis after extubation. In addition, the extubation criteria were as follows: oxygenation (PaO_2_ ≥ 100 mmHg, FIO_2_ ≤ 0.4, and positive end-expiratory pressure (PEEP) ≤ 4 cmH_2_O); clear consciousness level; normal temperature (> 35°C to < 38°C); and no use of excessive vasoactive agents.

In our ICU, NIV was performed according to a standardized procedure. Patients received NIV with a BiPAP Vision (Respironics, Murrysville, PA, USA) via a total face mask. The initial PEEP was 4–10 cmH_2_O, and subsequently adjusted to improve patient comfort. FIO_2_ was adjusted to achieve SpO_2_ of more than 94%, and if necessary, the pressure support was adjusted to maintain a tidal volume of 6–10 ml/kg. The head of the bed was raised at 30–45° during ventilation to minimize the risk of aspiration.

A clinical assessment (heart rate, arterial blood pressure, level of consciousness, SpO_2_, and respiratory rate) was performed regularly, as well as assessments of patient discomfort, air leaks around the mask, gastric distension, pressure sores, or facial skin necrosis. Arterial blood gases were tested prior to the initiation of NIV and then hourly for the first 2 h of NIV. Arterial blood gases were also tested when the patients were weaned from NIV or reintubated [8].

Successful NIV was defined as rapid improvement in the clinical status and gas exchange, with return to the earlier stable respiratory condition. Failure of NIV was defined as the need for endotracheal reintubation and mechanical ventilation during the ICU stay, irrespective of the reason.

We collected the following information: age; sex; risk factors including hypertension, diabetes mellitus, and renal replacement therapy if it was needed; Acute Physiology and Chronic Health Evaluation (APACHE) II score and Simplified Acute Physiology Score (SAPS) II at the start of NIV; reasons for AHRF; and outcomes including reintubation, ICU mortality, and in-hospital mortality. Arterial blood gases, heart rate, arterial blood pressure, and respiratory rate were recorded prior to the start of NIV, within 1, 2 and 6 h of the NIV start, and at discontinuation of NIV or reintubation. FIO_2_, pressure support, and PEEP were also recorded. We recorded the reasons for reintubation and renal replacement therapy if they were required.

In regard to reasons of AHRF, cardiogenic pulmonary edema was diagnosed if a patient had typical findings on chest radiographs, and widespread rales without a history suggesting pulmonary aspiration or infection, and high estimated right atrial pressure, and pulmonary artery pressures in echocardiography. Atelectasis was diagnosed if chest radiographs revealed a partial collapse of lung. Acute lung injury (ALI) was defined according to the American-European Consensus Conference on ARDS [9].

Statistical analysis was between the successful group and the reintubation group, or within-group over time. A two-way analysis of variance and the Bonferroni multiple comparisons procedure were performed to distinguish within-group differences over time. The Mann–Whitney test was performed to evaluate differences within the same time period and differences in the patient characteristics between the groups. Fisher’s exact test or the *χ*^2^ test was used to compare the reintubation rates, ICU mortality rates, and final in-hospital mortality rates, and to determine differences in the patient characteristics. The statistical analyses were performed using SPSS II (Abacus Concepts, Berkeley, CA, USA). All values are reported as means ± SD, and all values of *P* < 0.05 were considered to be statistically significant.

## Results

A total of 94 patients received NIV, of whom 89 patients (94%) successfully avoided endotracheal intubation (successful group) and five patients required reintubation (reintubation group) (Table 1). All patients, including the reintubated patients, were successfully weaned from mechanical ventilation and discharged from the ICU.

**Table 1:** Patient characteristics and factors related to NIV

	Total	Successful	Reintubation	*P* value
Number of patients (%)	94	89 (94%)	5 (6%)	
Age; years	67 ± 9	68 ± 9	63 ± 8	0.1995
Sex; female/male	30/64	29/60	1/4	0.5569
APACHE II score	15 ± 5	15 ± 5	18 ± 4	0.1793
SAPS II	28 ± 7	28 ± 7	30 ± 6	0.5717
Hypertension; n (%)	63	62 (70%)*	1 (20%)	0.0215
Diabetes mellitus; n (%)	38	36 (40%)	2 (40%)	0.9841
Renal replacement therapy; n (%)	20	19 (21%)	1 (20%)	0.9429
Reasons for AHRF; n (%)				
Cardiac pulmonary edema	58	56 (63%)	2 (40%)	0.3049
Atelectasis	26	24 (27%)	2 (40%)	0.5261
ALI	9	8 (9%)	1 (20%)	0.4155
Exacerbation of COPD	1	1 (1%)	0 (0%)	0.8117
Mode of NIV; n (%)				
CPAP	85	82 (92%)	3 (60%)	0.0586
Bilevel-PAP	9	7 (8%)	2 (40%)	0.0586
PEEP (cmH_2_O)	7 ± 2	7 ± 2	7 ± 2	0.8293
Pressure support (cmH_2_O)	4 ± 2	4 ± 1	4 ± 3	1
Time from hypoxemia to starting NIV (h)	2 ± 2	2 ± 2	3 ± 3	0.5394
Time from extubation to NIV (h)	20 ± 20	21 ± 21	8 ± 9	0.1638
NIV duration (h)	31 ± 24	32 ± 23	24 ± 37	0.1112

Data are shown as the mean ± SD unless otherwise indicated.

NIV, noninvasive ventilation; APACHE, Acute Physiology and Chronic Health Evaluation; SAPS, Simplified Acute Physiology Score; AHRF, acute hypoxemic respiratory failure; ALI, acute lung injury; COPD, chronic obstructive pulmonary disease; CPAP, continuous positive airway pressure; Bilevel-PAP, bilevel-positive airway pressure; PEEP, positive end-expiratory pressure.

*P < 0.05 vs. reintubation group.

Regarding the patient characteristics, there were no significant differences except for the numbers of patients with hypertension between the two groups (Table 1). Cardiogenic pulmonary edema was the major cause of AHRF, with atelectasis as the second most common cause (Table 1). There were no significant differences between the two groups in the reasons for AHRF. The successful rate of NIV was 97% for AHRF caused by cardiogenic pulmonary edema, which was higher than the average successful rate. On the other hand, the successful rate of NIV was lower for AHRF caused by atelectasis (92%) or acute lung injury (89%) compared with the average successful rate.

Overall, 90% of all AHRF patients were managed by CPAP. In both groups, the time from hypoxemia to starting NIV was within 3 h (Table 1).

In the reintubation group, two patients were reintubated as soon as NIV was started and we determined that NIV was ineffective. Two other patients were reintubated at 4 h and 16 h after the start of NIV, because PaO_2_/FIO_2_ improved within 2 h of NIV but then deteriorated, and respiratory discomfort reappeared. Although one other patient had successful NIV, the patient underwent tracheal reintubation after 51 h of NIV because of disquiet (Table 2). All the reintubated patients were successfully weaned from conventional mechanical ventilation and discharged from the ICU.

**Table 2:** Reintubation group

Case	Reasons for AHRF	Time from NIV to reintubation (h)	Reasons for reintubation	Length of intubation (h)	Surgical procedures
1	Cardiogenic pulmonary edema	0	Hypoxemia	36	DVR
					TAP
2	Cardiogenic pulmonary edema	4	Hypoxemia	14	CABG
3	Atelectasis	51	Disquiet	56	CABG
			Rejection of mask		Regeneration therapy
4	Atelectasis	0	Hypoxemia	87	DVR
					TAP
5	ALI	16	Hypoxemia	56	OPCAB
					Right lower lung lobectomy

AHRF, acute hypoxemic respiratory failure; NIV, noninvasive ventilation; ALI, acute lung injury.

DVR, double valve replacement; TAP, tricuspid annuloplasty; CABG, coronary artery bypass grafting; OPCAB, off-pump coronary artery bypass grafting.

Patients who underwent more than two surgical procedures tended to be reintubated more frequently than patients who underwent one surgical procedure. In the successful group, 24 patients (27%) underwent more than two surgical procedures, compared with four patients (80%) in the reintubation group.

No patients died in the ICU in both groups. Four patients (5%) in the successful group and one patient (20%) in the reintubation group died in the hospital (*P* = 0.1328). Their AHRF in the ICU improved, and they were managed under stable conditions in the general ward, and their hospital deaths had other causes.

PaO_2_/FIO_2_ at baseline was significantly higher in the successful group than in the reintubation group. In the successful group, PaO_2_/FIO_2_ improved and the respiratory rate decreased significantly within 1 h after the start of NIV. PaO_2_/FIO_2_ in the successful group was significantly better than in reintubation group during the whole NIV period. PaO_2_/FIO_2_ did not improve in the reintubation group (Table 3).

**Table 3:** Changes in hemodynamics, arterial blood gas values, and respiratory rates

	Group	Baseline	Within 1 h	Within 2 h	Within 6 h	Discontinuation of NIV or reintubation
Heart rate (bpm)	Successful	89 ± 15	87 ± 17	86 ± 15	86 ± 17	84 ± 15 ^*2^
	Reintubation	97 ± 19	96 ± 17	99 ± 12	95 ± 11	92 ± 10
Mean arterial blood	Successful	76 ± 15	77 ± 13	75 ± 13	77 ± 12	78 ± 11
pressure (mmHg)	Reintubation	77 ± 18	74 ± 8	74 ± 4	74 ± 8	74 ± 12
pH	Successful	7.44 ± 0.05	7.39 ± 0.17	7.44 ± 0.04	7.43 ± 0.12	7.45 ± 0.05
	Reintubation	7.40 ± 0.13	7.44 ± 0.05	7.40 ± 0.16	7.45 ± 0.07	7.40 ± 0.15
PaO_2_/FIO_2_ (mmHg)	Successful	143 ± 44 ^†1^	206 ± 79 ^*2, †1^	218 ± 66 ^*2, †1^	231 ± 72 ^*2,^^†2^	244 ± 67 ^*2, †2^
	Reintubation	103 ± 28	134 ± 79	139 ± 96	129 ± 58	103 ± 41
PaCO_2_ (mmHg)	Successful	37 ± 5	37 ± 5	38 ± 5	38 ± 4	38 ± 5
	Reintubation	45 ± 14	48 ± 22	47 ± 20	41 ± 4	48 ± 20
Respiratory rate	Successful	25 ± 6	23 ± 5 ^*2^	22 ± 5 ^*2^	21 ± 4 ^*2^	22 ± 4 ^*2^
(breaths/min)	Reintubation	25 ± 4	26 ± 6	26 ± 7	23 ± 10	23 ± 10

Data are shown as the mean ± SD.

NIV, noninvasive ventilation.

^*1^*P* < 0.05, ^*2^*P* < 0.01 vs. baseline.

^†1^*P* < 0.05, ^†2^*P* < 0.01 between the two groups.

## Discussion

In this study, we found that 94 patients with post-extubation AHRF after cardiovascular surgery received NIV, that 89 patients (94%) successfully avoided endotracheal intubation, and that NIV was effective for mild AHRF after cardiovascular surgery. All patients, including the reintubated patients, were successfully weaned from mechanical ventilation and discharged from the ICU. We think that it is important not to hesitate before performing reintubation when NIV is judged to be ineffective.

In this study, high successful rates of NIV for AHRF after cardiovascular surgery were observed because cardiogenic pulmonary edema was the major cause of AHRF, and atelectasis was the second most common cause. There is high quality evidence from meta-analyses and randomized trials that NIV decreases the need for intubation, and improves respiratory parameters, such as heart rate, dyspnea, hypercapnia, and acidosis, in patients with cardiogenic pulmonary edema [10]-13]. A meta-analysis of 13 trials (1,369 patients) found that patients who received CPAP plus standard care had a lower hospital mortality rate than those who received standard care alone [13]. Furthermore, the frequent incidence of atelectasis is a major concern after cardiovascular surgery, because it contributes to the deterioration of pulmonary function and oxygenation. The available evidence suggests that any type of lung expansion intervention is better than no prophylaxis. Incentive spirometry is the least labor-intensive technique, while CPAP is particularly beneficial for patients who cannot participate in incentive spirometry or deep breathing exercises [1]. In this study, AHRF caused by acute lung injury was present in a small number of patients. In a retrospective case–control study of 3,278 patients who underwent cardiac surgery and cardiopulmonary bypass, 13 patients (0.4%) developed acute respiratory distress syndrome during the postoperative period, and their mortality rate was 15% [14]. We should be aware of preventing severe hypoxemia after cardiac surgery and start treatment before severe hypoxemia occurs.

We used NIV for the early stage of mild AHRF after cardiovascular surgery, when it was noted from an initial PEEP of 7 cmH_2_O. The time from hypoxemia to starting NIV was within 3 h in both groups, and this early initiation of NIV led to high successful rates. In the successful group, PaO_2_/FIO_2_ improved and the respiratory rate decreased significantly within 1 h after initiation of NIV. The improvement of PaO_2_/FIO_2_ and the respiratory rate continued to be significant after 2 and 6 h of NIV and at discontinuation of NIV compared with the values before the start of NIV. PaO_2_/FIO_2_ in the successful group was significantly better than in the reintubation group during the whole NIV period. Two prospective studies showed that improvement of pH and PaCO_2_ within 0.5–2 h predicts the success of NIV [15,16]. In contrast, a patient should be considered to have NIV failure and be promptly intubated if there is neither stabilization nor improvement over the same time frame. Other criteria suggesting failure are worse encephalopathy or agitation, inability to clear secretions, inability to tolerate any of the interfaces, hemodynamic instability, or decreased oxygenation [15].

Although PaO_2_/FIO_2_ improved and the respiratory rate decreased significantly within 1 h after initiation of NIV in the successful group, NIV duration was 32 ± 23 h, and it seemed to be long. By necessity the discontinuance criteria of NIV was improvement of respiratory pattern and conservation of oxygenation level. NIV was the second mechanical ventilation after cardiovascular surgery, therefore in the clinical site we were careful about the weaning of NIV, and we decided to wean from NIV after we confirmed the hemodynamic sufficient stability and the enough improvement of water balance.

Engores et al. reported that reintubation rate after cardiac surgery was 4.1% [17]. In other study, 63 of 1,225 patients (5.1%) underwent NIV for respiratory failure after extubation after cardiac surgery, and reintubation was required in 52.4% of the NIV treated patients [18].

Five patients were reintubated among the study population. In the reintubation group, one patient had successful NIV, but underwent tracheal intubation after 51 h of NIV because of disquiet. The five patients were reintubated without any hesitation as soon as NIV was judged to be ineffective, and the length of intubation was 50 ± 27 h, which was a short time. All five patients were successfully weaned from conventional mechanical ventilation and discharged from the ICU. NIV appears to be ineffective and potentially harmful if it is not initiated until after the onset of post-extubation respiratory failure [5,6]. This was illustrated by a trial of 221 patients who developed respiratory failure after extubation and were randomly assigned to receive conventional medical therapy with or without NIV. All-cause mortality was increased in the NIV group, and the increased mortality may have been related to delayed reintubation [5]. NIV may be beneficial in preventing recurrent respiratory failure following extubation if employed at an early stage. Once a patient has been selected to receive a trial of NIV, it should be initiated as soon as possible. Patients who fail to improve or stabilize within 0.5–2 h should be promptly intubated, and we think that a motto of “do not hesitate to intubate” is important.

Although no patients died in the ICU in both groups, four patients (5%) in the successful group and one patient (20%) in the reintubation group died in the hospital. These patients were all weaned from mechanical ventilation, including NIV, recovered from AHRF, and were discharged from the ICU, and died of other causes. However, their durations of hospital stay were long, and the severity of the basic disease prolonged the hospital stay, which led to susceptibility to other complications.

This study had some limitations related to its design. It was a retrospective observational study in a single ICU with a small number of subjects. A large prospective study could provide conclusive evidence of the efficacy of NIV for AHRF after cardiovascular surgery. However, such a randomized controlled trial may be difficult because NIV has been widely used in postoperative critical care settings.

## Conclusions

We conclude that NIV is beneficial for AHRF patients after cardiovascular surgery when it is started at the early stage of mild AHRF, defined as PaO_2_/FIO_2_ ≤ 300 mmHg. We also think that it is important not to hesitate about reintubation when NIV is judged to be ineffective.

## Abbreviations

AHRF, Acute hypoxemic respiratory failure; NIV, Noninvasive ventilation; CPAP, Continuous positive airway pressure; ICU, Intensive care unit; PEEP, Positive end-expiratory pressure; APACHE II, Acute Physiology and Chronic Health Evaluation II SAPAS II: Simplified Acute Physiology Score II.

## Competing interests

The authors declare that they have no competing interests.

## Authors’ contributions

KN collected data, searched literature, and drafted the manuscript. ST planed the study, collected data, performed statistical analysis, searched literature, and drafted parts of manuscript. KT searched literature. AS searched literature. All authors read and approved the final manuscript.
